# Microarray-based selection of a serum biomarker panel that can discriminate between latent and active pulmonary TB

**DOI:** 10.1038/s41598-021-93893-3

**Published:** 2021-07-15

**Authors:** Zhihui Li, Jianjun Hu, Pengchong Liu, Dan Cui, Hongqin Di, Shucai Wu

**Affiliations:** Hebei Chest Hospital, Shijiazhuang, 050041 China

**Keywords:** Biomarkers, Diseases

## Abstract

Bacterial culture of *M. tuberculosis* (MTB), the causative agent of tuberculosis (TB), from clinical specimens is the gold standard for laboratory diagnosis of TB, but is slow and culture-negative TB cases are common. Alternative immune-based and molecular approaches have been developed, but cannot discriminate between active TB (ATB) and latent TB (LTBI). Here, to identify biomarkers that can discriminate between ATB and LTBI/healthy individuals (HC), we profiled 116 serum samples (HC, LTBI and ATB) using a protein microarray containing 257 MTB secreted proteins, identifying 23 antibodies against MTB antigens that were present at significantly higher levels in patients with ATB than in those with LTBI and HC (Fold change > 1.2; p < 0.05). A 4-protein biomarker panel (Rv0934, Rv3881c, Rv1860 and Rv1827), optimized using SAM and ROC analysis, had a sensitivity of 67.3% and specificity of 91.2% for distinguishing ATB from LTBI, and 71.2% sensitivity and 96.3% specificity for distinguishing ATB from HC. Validation of the four candidate biomarkers in ELISA assays using 440 serum samples gave consistent results. The promising sensitivity and specificity of this biomarker panel suggest it merits further investigation for its potential as a diagnostic for discriminating between latent and active TB.

## Introduction

Tuberculosis (TB), caused by *Mycobacterium tuberculosis* (MTB), is the leading cause of death from infectious diseases^1^. An estimated one-quarter of the world’s population is latently infected with MTB (latent tuberculosis infection (LTBI)), and 1.41 million people died from TB in 2019^2^. Approximately 5–10% of those infected with MTB will develop active tuberculosis (ATB) during their lifetime. Pulmonary infection accounts for 75% of active TB disease^3^ and is also the main factor underlying high tuberculosis mortality, especially in populations, such as individuals living with HIV-AIDS, with lowered immunity^4^. The identification of MTB-infected individuals and the appropriate treatment of those who develop ATB are undoubtedly crucial for effective TB control^5^.

Current clinical diagnosis of TB still relies on a traditional approach that includes acid-fast bacillus (AFB) smears, nucleic acid amplification (NAA) (e.g. Xpert MTB/RIF or Xpert MTB/RIF Ultra), and culture of *M. tuberculosis* from sputum and other respiratory specimens, in addition to evaluation of clinical symptoms^6^. However, sputum smears have very low sensitivity (10–20%), and MTB culture lacks sensitivity and requires 2–8 weeks to obtain results^7^. Although the speed and sensitivity of Xpert MTB/RIF is high, this technique cannot replace AFB sputum smears and culture methods due to its low sensitivity in detecting AFB-negative individuals, its high operation costs, and inconsistency in results between laboratories^8,9^. Prolonging diagnosis delays the commencement of appropriate treatment for TB; in the case of sputum smear-negative tuberculosis, delayed diagnosis is known to lead to irreversible lung injury^10^, one of the causes of TB’s high mortality. In vivo tuberculin skin tests (TST) and in vitro interferon gamma release assays (IGRAs) are considered auxiliary methods for diagnosing TB^11^; however, the TST cannot distinguish between healthy individuals vaccinated with the BCG and those with active TB disease. IGRA testing must be conducted under standard laboratory conditions by trained personnel^12^ and its reproducibility is disputed^13–15^. In addition, neither test can distinguish between ATB and LTBI^16,17^. There is therefore an urgent need for a rapid, simple and more accurate method for diagnosing active TB disease.

Serological screening of disease-related serum biomarkers is a convenient, quick and non-invasive method for disease diagnosis. The sensitivity and specificity of biomarkers for the disease in question is an important indicator of whether they can be used in clinical practice, determining the reliability of test results^18^. Serum biomarkers for TB, such as the 38kD, 16kD, ESAT-6, MPT63, 19kD, MPT64, MPT32, Rv1009, MTB48, Mtb81, MTC28, Ag85B, and KatG proteins have been identified^19^, but while most show high sensitivity in ATB patients^20^, their sensitivity and specificity is insufficient for discriminating between active TB and LTBI^21^. The screening of new TB serum biomarkers that can discriminate between active TB and LTBI is thus of great importance for improving diagnostic accuracy.

Here, we set out to identify serum biomarkers that can be used to diagnose active pulmonary TB. Reasoning that secreted proteins are likely to be a useful source of biomarkers, we used an MTB protein microarray containing 257 *M. tuberculosis* recombinant secreted proteins. In the biomarker discovery phase of the study, we screened 116 samples (52 ATB, 37 LTBI, and 27 HC) with the microarray, identifying 23 antibodies that showed a differential pattern between the ATB and LTBI and HC groups. We then identified a panel of 4 proteins with high sensitivity and specificity that could distinguish ATB from LTBI, and verified the performance of these four proteins using ELISA assays and 440 serum samples. This panel merits further investigation for its potential in diagnosing active pulmonary tuberculosis.

## Materials and methods

### Study cohort

Serum samples from individuals with active tuberculosis (ATB, 205) or latent tuberculosis (LTBI, 123) and healthy donors (HC, 112) included in this study were collected at Hebei Chest Hospital between May 2018 and Jan 2019. Diagnosis of active tuberculosis was based on standard criteria, including clinical symptoms, chest radiograph abnormalities, AFB sputum smears, bacterial culture and IGRA (X.DOT-TB, TB Healthcare, Foshan, China) results. The active TB group included individuals who displayed TB-specific clinical symptoms, had abnormal chest radiography consistent with active TB, and were sputum AFB positive and/or bacterial culture positive. Samples from patients in the ATB group were collected before treatment. The LTBI group included individuals who did not display clinical symptoms, but were IGRA positive and showed no signs of active TB in chest X-rays. The healthy control (HC) group included individuals who did not display clinical symptoms, and were IGRA negative and showed no signs of TB in chest X-rays. Individuals who tested positive for human immunodeficiency virus (HIV), or were taking immunosuppressive or immunopotentiator agents were excluded from the study. This study was approved by the Ethics committee of Hebei Chest Hospital (Hebei Province, China), in accordance with the Declaration of Helsinki (No. 2020076). Informed consent was obtained from all subjects or their legal guardians if the subjects were under 18.

### Serum samples

Peripheral blood samples (5 mL) were collected in vacutainer tubes. Sera were obtained by centrifugation at 1509×*g* for 10 min, and were then aliquoted into sterile polypropylene microtubes and stored at − 80 °C until required.

### Profiling on MTB secreted protein microarrays

The MTB proteome microarrays used in this study were constructed by BC-BIO (Guangzhou, China). Microarrays comprised 257 recombinant *M. tuberculosis* H37Rv secreted proteins. All proteins had GST tags and were expressed and purified using a *S. cerevisiae* expression system. Microarrays were incubated with GST antibodies to assess their quality, human IgG and IgM being used as positive controls, and bovine serum protein (BSA) as a negative control.

Microarrays were blocked in blocking buffer (1× PBS, 3% BSA, 0.1% Tween 20 [pH 7.4]) for 3 h at room temperature with shaking. After blocking, 200 μL serum samples (1:50 dilution in PBST-B (1× PBS, 1% BSA, 0.1% Tween 20 [pH 7.4])) were overlaid onto protein microarrays and incubated at 4 °C overnight. After washing three times in PBS containing 0.1% Tween 20 detergent (PBST), arrays were probed with goat anti-human IgG conjugated with Cy3, and goat anti-human IgM conjugated with Cy5 (Jackson Laboratory, PA, USA) at a 1:1000 dilution and incubated in a dark room for 1 h at room temperature. After washing three times in PBST and twice in ddH_2_O, arrays were dried in a SlideWash (Capital Bio) and then scanned in a GenePix 4200A slider scanner (Molecular Devices).

### Protein array data preprocessing

The median values of the foreground (Fij) and background (Bij) intensities of a given protein spot (i,j) on the protein array were extracted from quantified sample array image data exported from GenePix Pro 6.0. The ratio of the mean signal (protein spot signal intensity (Rij): Fij/Bij) over the mean background signal was determined for each protein spot. This preprocessing method thus normalized all features in the array to their background signals. As each protein was printed on the array in duplicate, Rij was averaged for each protein as Rp.

### Data analysis

SAM (Significance analysis of microarrays, R software (v3.6.1))^22^ was used to determine proteins to which ATB, LTBI and HC samples showed a statistically significant immunogenic response. Stringent criteria were set; significantly upregulated proteins were only called if the ratio of signal intensity between the two groups being compared was > 1.2 at *p* ≤ 0.05. Significantly downregulated proteins were called if the ratio of the signal intensity values was < 0.83. The heatmap for the differential protein set was drawn using the ‘pheatmap’ package (v1.0.12)^23^ in the R statistical language (v3.6.1)^22^. Biomarker candidates were the differential proteins with the highest discriminant ability (Discriminant ability = Sensitivity + Specificity − 100%)^24^ and were selected for each comparison (ATB versus LTBI and ATB versus HC). Candidate biomarkers were differential proteins (fold change > 2 and *p*-value < 0.05) that satisfied the following criteria: (1) they showed at least 90% specificity and (2) had the highest discriminant ability (> 10%).

### Functional analysis

GO (Gene Ontology) classification of differential proteins according to molecular function and biological process was based on their functional annotations using the R package clusterProfiler (v3.4.4)^25^. GO ‘Biological Process’ (BP)^26^ and ‘molecular function’ (MF) enrichment analyses were also performed for the two groups of differential proteins using clusterProfiler. The *p*-value threshold for significance was selected as < 0.05. Protein–protein interactions (PPI) between differential proteins were analysed using the STRING database (v10.0)^27^, and networks were visualized with Cytoscape (v3.4.0)^28^.

### ELISA assays

MTB antigens (BCBIO, Guangzhou, China) were diluted to 1 μg/mL with a coating solution (sodium carbonate 1.59 g/L, sodium bicarbonate 2.93 g/L), added to 96-well plates, and allowed to stand overnight at 4 °C for coating. Plates were washed three times with PBST, then blocked with blocking buffer (1× PBS, 3% BSA, 0.1% Tween 20 [pH 7.4]) at room temperature for 3 h. 100 μL serum samples (diluted 1:100 in PBST-B) were added to antigen-coated wells and incubated at room temperature for 30 min. After washing the plates five times with PBST, anti-human IgG antibody (CWBiotech, Beijing, China), diluted 1:10,000 in PBST-B, was added, and incubated at room temperature for 30 min. After washing five more times with PBST, plates were developed using TMB substrate (BD, NJ, USA) in a dark room for 10 min at 37 °C; reactions being stopped using 2 mol/L sulfuric acid. The optical density of the wells was determined at 450 nm/620 nm using an automated microplate reader (Perlong, Beijing, China).

### Statements on study approvals

We confirm that all methods used in this study were carried out in accordance with relevant guidelines and regulations.

## Results

### Overall study design

**Figure 1:**
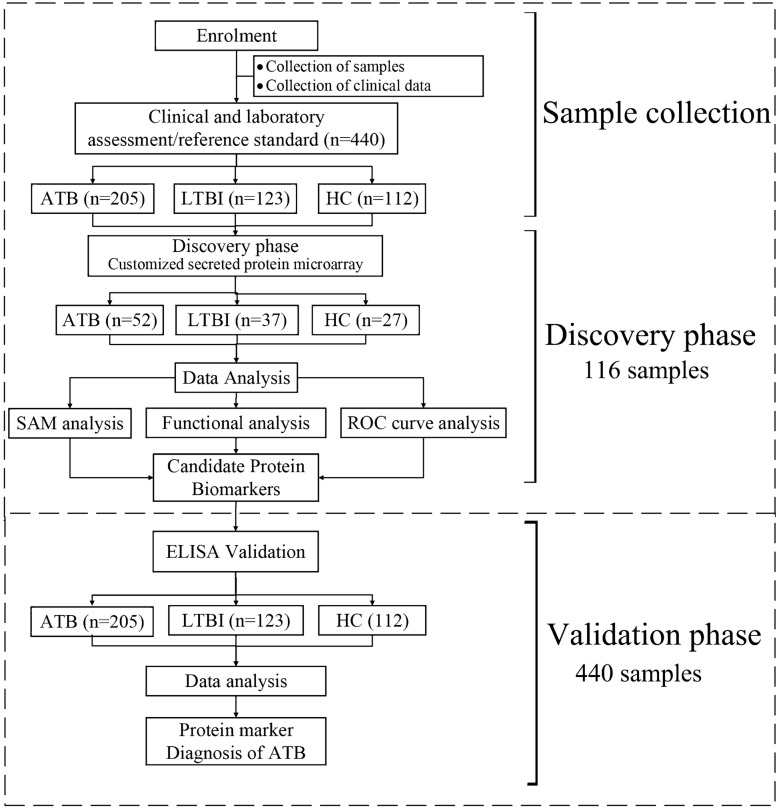
Identification of serum biomarkers that can discriminate between ATB and LTBI/healthy groups. Overall study design.

We employed a two-phase strategy^29,30^ to identify novel biomarkers for ATB (Fig. 1). In the discovery phase, we profiled serum samples from 52 ATB patients, 37 individuals with LTBI and 27 healthy volunteers on a protein microarray containing 257 *M. tuberculosis* secreted proteins. After identifying differential antigens, i.e. proteins present at different levels in samples from ATB patients and those from individuals with LTBI or healthy individuals, promising candidate biomarkers were selected based on their sensitivity and specificity in the differential diagnosis of ATB. A panel of four biomarkers that gave optimal sensitivity and specificity was then selected using SAM (significance analysis of microarrays). In the subsequent validation phase of the study, an additional 324 serum samples were evaluated in a separate ELISA experiment along with the 116 samples used in the discovery phase (205 ATB, 123 LTBI, 112 HC; Table 1; Fig. 1).

**Table 1:** Demographic and diagnostic information for the study cohort.

	Active tuberculosis	Latent tuberculosis	Healthy donors
**Discovery phase**			
Number	52	37	27
Average age (range)	40.3 (15–76)	36.1 (17–84)	39 (18–91)
Gender (M/F)	34/19	21/16	11/16
Smear-positive (%)	28 (53.8%)	–	–
Culture-positive (%)	37 (71.1%)	–	–
IGRA positive	–	37	0
**Validation phase**			
Number	205	123	112
Average age (range)	41.9 (15–90)	39.5 (17–84)	33.5 (18–91)
Gender (M/F)	111/94	70/53	42/70
Smear-positive (%)	102 (49.7%)	–	–
Culture-positive (%)	150 (73.2%)	–	–
IGRA positive	–	123	0

### MTB secreted protein microarrays

In our search for serum biomarkers associated with active TB, we focused on secreted proteins, reasoning that they mediate important functions through their interactions with host cells such as macrophages and are thus likely important in virulence and pathogenesis^31,32^. We thus used a protein microarray containing 257 secreted proteins from the *M. tuberculosis* reference strain H37Rv^32,33^ to profile serum samples from people with differing TB status. We first tested the quality and stability of the microarrays by profiling one sample from the ATB, LTBI, and HC groups (selected randomly) on secreted protein microarrays. Microarray quality was validated by comparing the signal intensity of the two replicate points for each protein on each array (Supplementary Fig. S1). The average Pearson correlation coefficient (R^2^) between replicate proteins was 0.99 (Supplementary Fig. S1, top right), indicating that the secreted protein microarrays were homogeneous and reproducible.

### Differential antibodies identified using the secreted protein microarray

**Figure 2:**
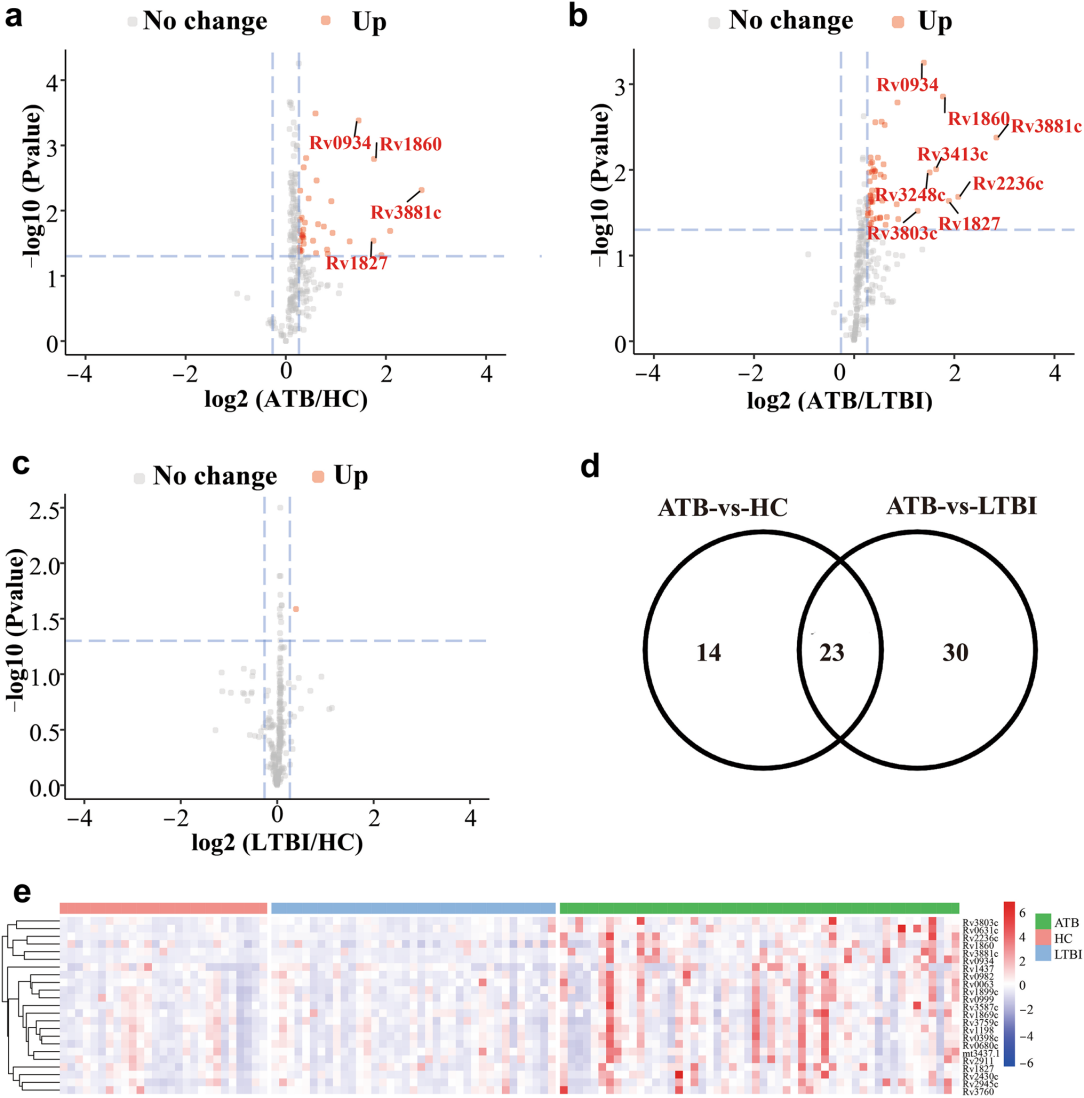
Microarray-based selection of antigens present at different levels in serum samples from individuals with active TB or LTBI or healthy controls. (**a–c**) Volcano plots. Differential analysis of serum samples from the ATB and LTBI groups (**a**), ATB and HC groups (**b**) and the LTBI and HC groups (**c**). Volcano plots show the change in transformed p-value (− log_10_) against the log_2_ ratio in the three groups of serum samples. Blue dashed lines: cut-off values (ratio > 1.2 and p-value < 0.05). Red dots: antigens present at increased levels. Important proteins present at increased levels are labeled. (**d**) Venn diagram. Twenty-three antigens present at higher levels in the ATB group were present in both the ATB-vs-HC and ATB-vs-LTBI comparisons. (**e**) Hierarchical clustering of the 23 differential proteins (from (**d**)) in the ATB, LTBI and HC groups. Color bar: signal intensity of the antigen. The heatmap was drawn using the ‘pheatmap’ package (v1.0.12)^23^.

In the discovery phase, 52 ATB, 37 LTBI, and 27 HC serum samples were profiled on MTB secreted protein microarrays, antibodies against MTB secreted proteins on the microarray being identified by developing the arrays with Cy3-labeled anti-human IgG antibodies. Signal intensities were normalized against the median value of the microarray, and comparisons were made between the groups to identify differential antibodies, thresholds being set at *p* ≤ 0.05 and fold change > 1.2. Using these criteria, 37, 53, and 1 differential proteins were identified in the ATB-vs-HC, ATB-vs-LTBI, and LTBI-vs-HC comparisons respectively (Fig. 2a–c).

Cluster analysis of the differential proteins showed that the ATB group was well separated from the HC and LTBI groups (Supplementary Fig. S2). The lists of differential proteins identified in the ATB-vs-HC and ATB-vs-LTBI comparisons had 23 proteins in common. These 23 proteins are likely a source of biomarkers that can discriminate the ATB group from the HC and LTBI groups (Fig. 2d,e).

### Gene Ontology and protein–protein interaction network analysis

**Figure 3:**
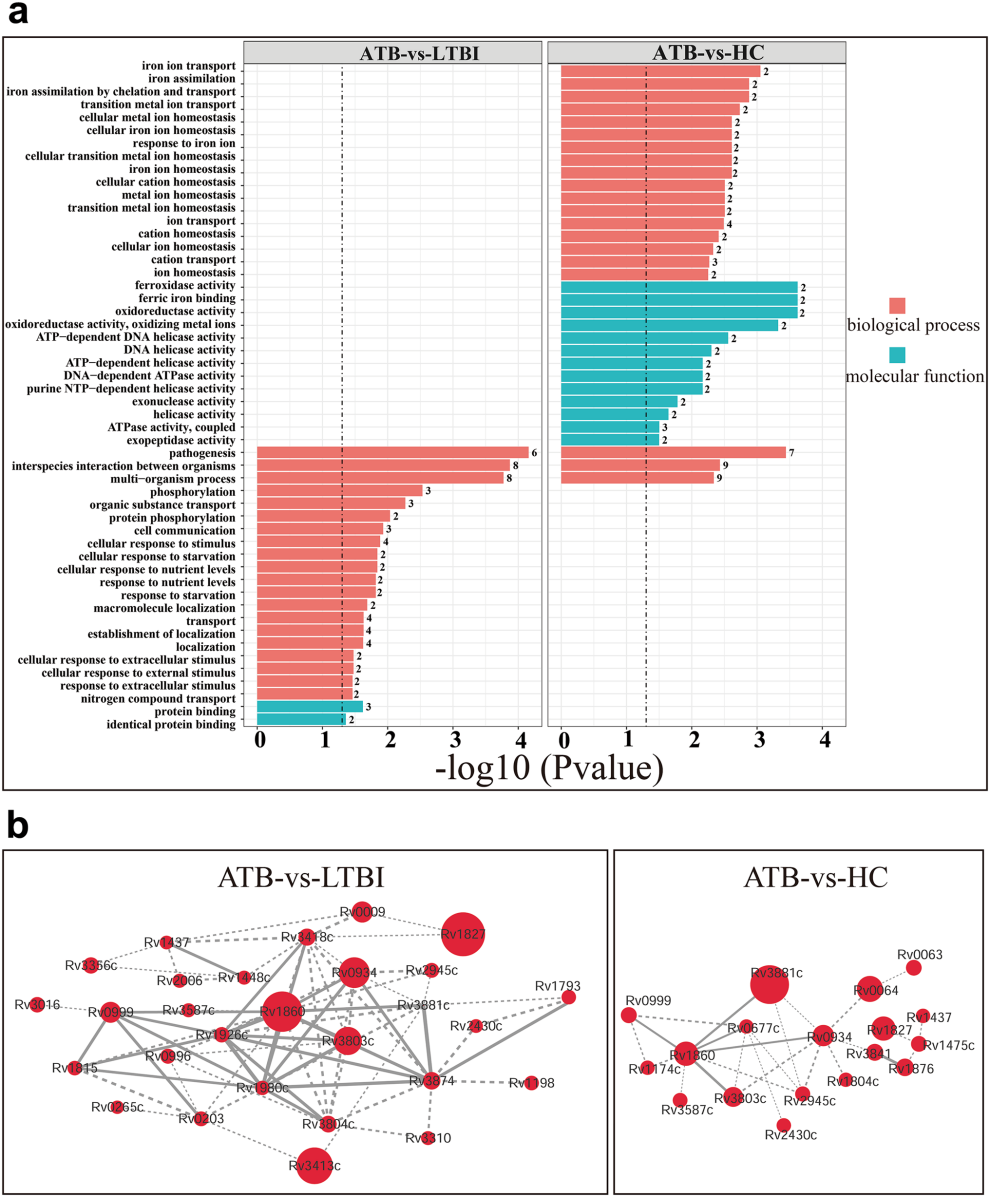
Data enrichment analysis of differential proteins according to molecular function and biological process using Gene Ontology. (**a**) Gene Ontology enrichment of differential antigens according to biological process (red) and molecular function (green). Y-axis: name of the enrichment GO term, X-axis: transformed P-values (−log_10_) (threshold: 0.05). Left panel: ATB-vs-LTBI, right panel: ATB-vs-HC. (**b**) Network analysis of differential proteins. The protein–protein interaction networks of differential proteins were analyzed by STRING, and the network was visualized using Cytoscape. The minimum required interaction score was set as the highest confidence (0.400) and the disconnected nodes in the network were hidden. Node size: the degree of difference (the larger the circle, the greater the ratio of ATB/HC or ATB/LTBI). Dashed line: interaction score < 0.7, solid line interaction score > 0.7. Left panel: ATB-vs-LTBI, right panel: ATB-vs-HC.

Data enrichment analysis was performed using clusterProfiler (R package) and GO annotation, and differential antigens were considered to be significantly enriched in a particular GO term when p < 0.05. Differential proteins from the ATB-vs-HC comparison were enriched in the ‘molecular function’, ‘ferroxidase activity’, ‘ferric iron binding’, and ‘oxidoreductase activity’ terms, while those from the ATB-vs-LTBI comparison were enriched in the ‘protein binding’ term (Fig. 3a green bar chart).

Biological process enrichment analysis of differential proteins identified in the ATB-vs-LTBI comparison indicated that processes related to interspecific interactions showed significant enrichment (Fig. 3a). In the ATB-vs-HC comparison, differential proteins were significantly enriched in biological processes related to the chelation and transport of metal ions (*p* < 0.05), the three most enriched biological processes being pathogenesis, iron ion transport, and iron assimilation (Fig. 3a).

We used STRING analysis of protein–protein interaction networks to determine which differential proteins from the ATB-vs-LTBI and ATB-vs-HC comparisons were important in the functional network. Key proteins included Rv1827, Rv1860, Rv3413, Rv0934, Rv3803c, and Rv3881c (ATB-vs-LTBI comparison; Fig. 3b left panel), and Rv3881c, Rv0064, Rv1827, Rv1860, and Rv0934 (ATB-vs-HC comparison; Fig. 3b right panel). Although the biological process and molecular function enrichment results for the ATB-vs-HC and ATB-vs-LTBI comparisons were different, there was some overlap in key proteins, Rv3881c, Rv1860, Rv0934, and Rv1827 being common to both lists.

### ROC analysis of biomarkers and derivation of a combined panel for ATB diagnosis

**Figure 4:**
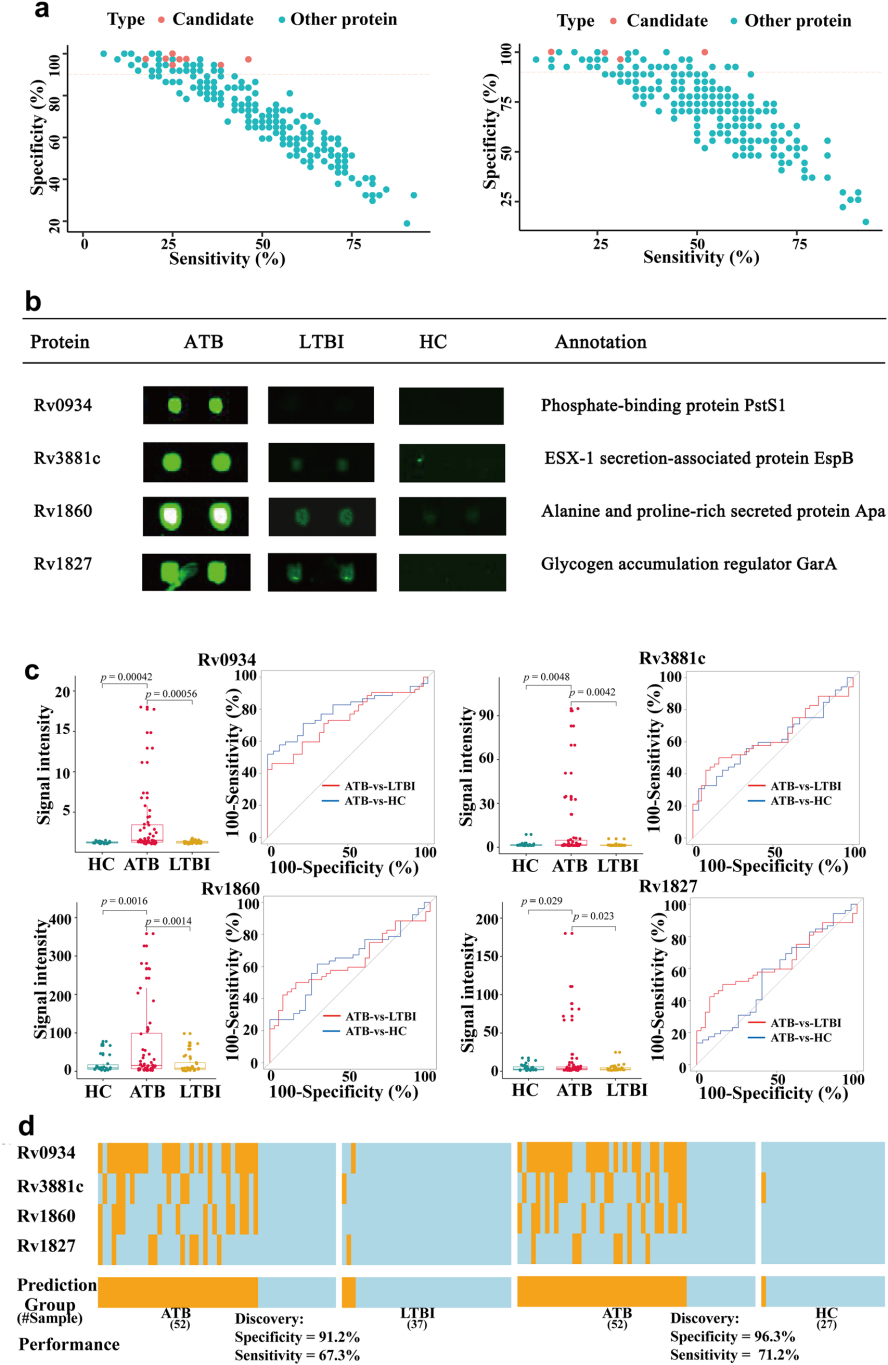
Biomarker discovery. (**a,b**) Scatter plots of the sensitivity and specificity of all proteins on the microarray. Each dot represents one protein. Left panel: proteins present at different levels in the ATB-vs-LTBI (left panel) and ATB-vs-HC (right panel) comparisons. Red dots: proteins with (1) a discriminant ability (sensitivity + specificity − 100) > 10%; (2) a fold change > 2 and a p-value < 0.05; and (3) at least 90% specificity. (**b**) Microarray results and annotations of the 4 proteins selected as candidate biomarkers. (**c**) Box plots and ROC curves for each of the 4 candidate biomarkers. Left panels: box plots show that the signal intensities of the each candidate biomarker is significantly higher in the ATB group than in the HC and LTBI control groups. Right panel: ROC curves. The sensitivity and specificity values obtained at the optimal cut off value for each candidate biomarker are also shown. (**d**) Performance of the top biomarker panel, comprised of Rv0934, Rv3881c, Rv1860, and Rv1827. Orange and light blue lines represent samples scored as positive or negative, respectively. A sample was predicted as ATB positive when any of the four proteins in the panel was positive. The heatmap was drawn using the ‘pheatmap’ package (v1.0.12)^23^.

To identify potential serum biomarkers for active pulmonary TB diagnosis, we plotted receiver operating characteristic (ROC) curves for differential proteins and calculated the areas under the curve (AUCs). Sensitivity and specificity values were calculated for each protein. Selecting the following criteria (a) specificity > 90%; (b) discriminant ability (sensitivity plus specificity − 100) > 10%; (b) fold change > 2; and (c) statistical significance *p* < 0.05), resulted in identification of 8 candidate proteins from the anti-IgG profiles in the ATB-vs-LTBI comparison and 4 candidate proteins from the anti-IgG profiles in the ATB-vs-HC comparison, respectively (Fig. 4a,b, Supplementary Table S1, S2). The signal intensities of the four proteins common to both the ATB-vs-LTBI and ATB-vs-HC comparisons, Rv0934, Rv3881c, Rv1860 and Rv1827, were all significantly different (Fig. 4c). The diagnostic performance of each candidate biomarker was assessed using areas under the receiver operating characteristic (ROC) curves (AUCs). The AUC values of the four proteins ranged from 0.56 to 0.788 (Table 2). Next the maximum discriminant ability values for each protein were calculated with a requirement of a minimum specificity of 90% (see “Materials and methods” section). The optimal cutoff values of the signal intensity for each protein were then determined with the corresponding sensitivity and specificity values (Table 2).

**Table 2:** AUC analysis of potential ATB diagnostic biomarkers in the ATB-vs-LTBI and ATB-vs-HC comparisons.

Protein^a^	ATB-vs-LTBI			ATB-vs-HC		
	AUC	Specificity (%)	Sensitivity (%)	AUC	Specificity (%)	Sensitivity (%)
Rv0934*	0.735	97.297	46.154	0.788	100.000	51.923
Rv1827*	0.560	97.297	17.308	0.563	100.000	13.462
Rv1860*	0.655	100.000	25.000	0.637	100.000	26.923
Rv3881c*	0.634	97.297	23.077	0.611	96.296	30.769
Rv2236c	0.708	97.297	28.163	0.710	77.778	63.462
Rv3248c	0.680	94.595	25.000	0.491	59.259	53.846
Rv3413c	0.629	97.297	26.923	0.570	88.889	32.692
Rv3803c	0.674	94.595	38.462	0.670	48.148	82.692

^a^Proteins marked with an asterisk (*) were selected as candidate biomarkers in both comparisons.

Of note, proteins Rv0934, Rv3881c, Rv1860 and Rv1827 had sensitivities > 46.2% and specificities > 97.3%, making them very good candidates for diagnostic biomarkers.

We noted that the sensitivity values of each candidate protein ranged from 13.46 to 46.15%, so attempted to identify a combined biomarker panel that would offer better performance. The performance of all possible combinations (14 combinations) of between two and four proteins was evaluated using a previously reported approach^34^. Firstly a binary scoring system was used to convert the actual signal intensity of each protein to either 1 or 0, with 1 represented a signal intensity greater than the optimal cutoff value and 0 represented a signal intensity lower than the optimal cutoff value. Secondly the performance of every possible combination in the discovery cohort was evaluated. The total binary score of a given combination of n proteins was assigned to each serum sample as a summary score. If the summary score of a sample was greater than k (1 ≤ k ≤ n), the sample was determined as positive. For each protein combination, the sensitivity and specificity at the best discriminant ability were recorded. Finally, the optimal protein combination and its k value with the best discriminant ability at a minimum specificity of 90% were identified. The best panel combination, comprised of Rv0934, Rv3881c, Rv1860 and Rv1827, achieved 67.3% sensitivity at 91.2% specificity with a k value of 1 for discriminating ATB from LTBI, and 71.2% sensitivity at 96.3% specificity for discriminating ATB from HC (Fig. 4d). In other words, a serum sample would be scored positive when at least one (i.e. k = 1) of the four proteins showed a signal intensity greater than the corresponding optimal cutoff value. These promising high sensitivity and specificity values suggest that this panel of four proteins is worth investigating further and applying in the development of a diagnostic panel for ATB.

### Validation of the ATB biomarker panel using ELISA assays

**Figure 5:**
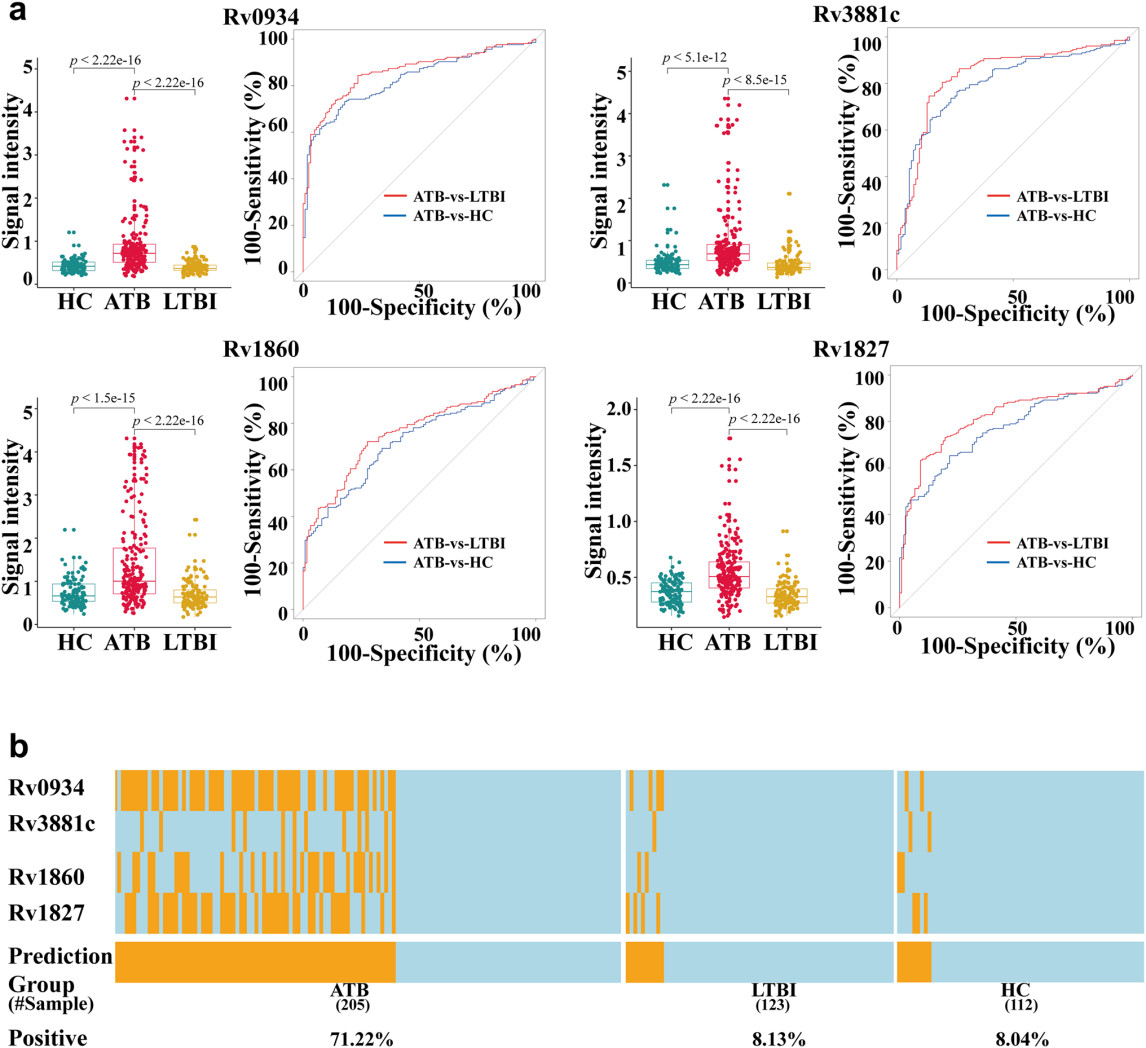
ELISA validation of candidate biomarkers. (**a**) Validation of the 4 candidate protein biomarkers. Left panels: box plots showing that the signal intensities of the four candidate biomarkers are significantly higher in the ATB group than in the HC and LTBI control groups. Right panels: ROC curves. The sensitivity and specificity values obtained at the optimal cut off value are shown for each protein. (**b**) Performance of the top biomarker panel, comprised of Rv0934, Rv3881c, Rv1860, and Rv1827. Orange and light blue lines represent samples scored as positives and negatives, respectively. A sample was predicted as ATB positive when any of the four proteins in the panel was positive. The heatmap was drawn using the ‘pheatmap’ package (v1.0.12)^23^.

To develop a clinically useful application of the biomarkers identified here, we verified the biomarkers using an enzyme-linked immunosorbent assay (ELISA) platform. Rv0934, Rv3881c, Rv1860, and Rv1827 were purified from yeast and coated onto the individual wells of ELISA plates. Results obtained were consistent with those obtained in the discovery phase using microarrays; all four proteins showed significantly higher signals in the ATB group than in the HC or LTBI groups (Fig. 5a, Supplementary Table S3). When specificity was set at > 90%, the four proteins had sensitivities ranging from 13.17 to 59.02%, and ROC analysis of ELISA data gave AUC values for the four proteins of between 0.76 and 0.86.

We next evaluated the performance of the biomarker panel against the ELISA data. The ELISA data were converted to a binary scoring system in a manner similar to that in the discovery phase, and the same criteria (specificity > 90%) were used. 71.22% and 66.83% of ATB samples in the ATB groups were correctly scored as positive in the ATB-vs-LTBI and ATB-vs-HC comparisons, respectively (Fig. 5b), while only 8.04% of samples in the LTBI group and 8.12% of samples in the HC group were scored as false positives. This biomarker panel thus showed a sensitivity of 71.22% at a specificity of 91.87% for ATB diagnosis based on ELISA tests.

## Discussion

The lack of a rapid diagnostic test for ATB hinders the timely diagnosis and treatment of this devastating infectious disease, impacting patient management and ultimately resulting in further disease transmission. Here, by performing a systematic search for secreted MTB antigens that can distinguish ATB from LTBI using an MTB secreted protein microarray and serum samples, we identified a four antigen panel (Rv0934, Rv1827, Rv1860 and Rv3881c) that could distinguish ATB from LTBI/healthy individuals with a sensitivity of 67% and specificity of 92%. Validation of this protein panel by ELISA gave similar results. This ATB diagnostic panel deserves further evaluation with larger cohorts of ATB and LTBI samples from different locations to determine its broad applicability.

The considerable efforts invested in developing tests that can distinguish latent and active TB have yet to yield a test with sufficient discriminatory power for clinical application. The approach taken in most studies has been to evaluate the discriminatory ability of known MTB antigens or combinations of antigens, rather than to take a systematic approach to antigen discovery. MTB antigens identified so far that can discriminate individuals infected with MTB from those without infection (but not from those with active TB) include ESAT-6 (Rv3875), CFP-10 (Rv3874), Ag85B (Rv1886c), LAM, Mtb81, MTC28, and KatG^35,36^. Antigens evaluated for their ability to differentiate latent from active TB have been reviewed in detail elsewhere^37^, and include latency-associated antigens from the Dormancy of survival Regulon (DosR, including Rv0081, Rv1733, Rv1737, Rv2029c, and Rv2031), and the Region of Difference (Rv2659c, Rv2660c), Resuscitation promoting factors (Rpfs, Rv0867c, Rv2389c), reactivation associated antigens (Rv1131 and Rv3862c) and the Ag85 complex (Ag85A, Ag85B and Ag85C). While many studies have been performed, to date, a panel of biomarkers with adequate sensitivity and specificity for discriminating LTBI and active TB has been elusive.

Here, we systematically searched for secreted proteins with high discriminatory ability, reasoning that these key components of the MTB weaponry that play important roles in pathogenesis, secreted at different stages of infection, likely lead to variation in the host immune response^31,32^ and are thus a reservoir of potential biomarkers for discriminating different stages of disease. MTB can remain dormant within its host for a long time, interactions between secreted bacterial proteins and host cells mediating its ability to evade the host immune system and survive within macrophages.

Development and application of high-throughput technologies for screening serum biomarkers of various diseases has shown great promise for facilitating disease diagnosis^38^. Protein microarray technology can be used to detect changes in large numbers of proteins within a sample simultaneously, and can detect changes in the modification status of proteins, making it especially suitable for screening disease diagnostic markers and drug targets. Here, we used a commercial MTB secreted protein microarray containing 257 secreted proteins from the H37Rv reference strain purified using a yeast expression system^32,33^. Proteins expressed using a yeast system have posttranslational modifications^39^ and are suitable for screening functional antigens^40^. Our use here of a microarray using yeast-expressed proteins rather than proteins purified using the *E. coli* system may have facilitated our discovery of previously unreported antigens associated with active TB disease.

Our study design has several strengths. First, we employed a commercial TB secreted protein microarray with a high coverage of TB secreted proteins to improve the likelihood of finding potential biomarkers. Second, our study included samples from 328 patients with ATB or LTBI and 112 samples from healthy volunteers. Third, we used samples from both LTBI and healthy individuals as negative controls to improve discrimination of ATB. Finally, we used ELISA as an independent platform to validate the performance of the newly discovered biomarker panel.

Individual antigens rarely perform well enough to be useful as single biomarkers for a disease, but a panel of several markers can significantly improve performance. Here, individual antigens ranged in sensitivity from 17 to 46%, but when four promising antigens were combined into a panel, the sensitivity of the panel was 62% at a specificity of 92%. Consistent levels of antigen-specific IgG antibodies were detected in ELISA experiments to validate these four antigens, all showing significant differences between healthy individuals, those who had LTBI, and those who had active TB, suggesting that these antigens may elicit strong humoral immune responses.

To the best of our knowledge this is the first time that these biomarker candidates have been reported in a marker panel for discriminating LTBI from active TB. Interestingly, previous reports on each of these biomarker candidates link them with different aspects of virulence or pathogenesis. Rv0934 (Periplasmic phosphate-binding lipoprotein, PstS1) is a 38-kDa antigen commonly used in tests for diagnosing TB infection^41^. On its own, however, Rv0934 does not have sufficient sensitivity to discriminate active from latent TB. It belongs to the family of phosphate receptors for bacterial ABC-type lipoprotein transporters and is involved in active binding-protein-mediated import of inorganic phosphate across the membrane^42^. Rv1827 (GarA) is a conserved essential protein containing an FHA domain in its C-terminus. It is phosphorylated by Serine/threonine protein kinase G (PknG), an important virulence factor, and participates in the regulation of the tricarboxylic acid cycle^43^. Rv1860 (Alanine and proline rich secreted protein Apa) is a 45-kDa secreted glycoprotein predicted to be involved in cell wall and cell processes. It is a major immunodominant antigen that is considered to have potential as a vaccine against tuberculosis, and can elicit proliferation of both CD4+ and CD8+ T cells and IFN-γ secretion in healthy people with LTBI^44^. Changes in the mannosylation pattern of this protein affect its ability to stimulate T-lymphocyte responses. Virulence factor Rv3881c (Secreted ESX-1 substrate protein B, EspB) is a conserved alanine and glycine rich protein that is a member of the PE-PPE family^45^. It is reported to inhibit autophagosome formation in murine macrophages via downregulating the expression of IFN-γ receptor 1^46^. Further mechanistic research on the involvement of these candidate biomarkers in the pathogenesis of active TB may provide interesting insights into the pathogenesis of active TB.

Our study also has some limitations. By focusing on secreted proteins, we may have missed other cell wall, or indeed intracellular proteins that provoke an important immune response during active disease. The number of samples tested in our study (440), particularly in the discovery phase (116) was limited. While the panel identified here showed high discriminatory power in this study cohort, one limitation is that all participants recruited were Chinese, raising the small possibility that there could be an ethnic bias. A larger cohort of samples, preferably drawn from more than one geographical region will be necessary to further evaluate the potential of this panel as a diagnostic test. We also did not discriminate between AFB positive and AFB negative active TB cases, nor did we consider extrapulmonary TB cases. All patients included were adults, so we are unable to predict if this panel would be efficacious for the diagnosis of childhood TB. In addition, pulmonary TB patients included in our study were sputum AFB positive and/or bacterial culture positive cases. The specificity of our marker panel for distinguishing ATB from HC in such cases was high (96.3%), however, its sensitivity (71.2%) falls short of that specified in the WHO’s optimal Target Product Profile (TPP) for non-sputum-based TB diagnosis tests (98%)^47^. Nonetheless, like other biomarker studies published that similarly did not reach these standards^9,48,49^, our study has useful reference value for those engaged in biomarker discovery. Further work on optimizing this panel will be necessary.

Of note, our four-antigen panel was unable to discriminate between individuals with latent TB and uninfected individuals (control group). Given the availability of many commercial platforms for IGRA-based detection of MTB infection based on ESAT-6/CFP-10-induced stimulation of IFN-γ release, the most likely application of the biomarker panel developed here would be as an ELISA test that is combined with one of the commercial ESAT-6/CFP-10 IGRA assays. Further research is also needed to identify biomarkers associated with other stages of MTB infection (e.g. recovery, re-activation), and prognostic markers that can predict treatment outcome.

The availability of rapid tests that can accurately detect active TB using the biomarker panel developed here should not only facilitate timely treatment of patients with TB, but should also greatly facilitate the implementation of proactive TB case-finding programs and ultimately reduce the spread of TB within the community, making a significant contribution to achieving the WHO’s End TB goals for eliminating TB by 2035.

## Supplementary Information

Supplementary Figures.

Supplementary Tables.
