# Supplementary material for: Microarray-based selection of a serum biomarker panel that can discriminate between latent and active pulmonary TB
Source: Sci Rep. 2021 Jul 15;11:14516. doi: 10.1038/s41598-021-93893-3 (PMC8282789; doi:10.1038/s41598-021-93893-3)
Supplement: Supplementary file 1 — Supplementary Figures. [file 41598_2021_93893_MOESM1_ESM.pdf]

Microarray-based selection of a serum biomarker panel that can discriminate between latent and active pulmonary TB

Zhihui Li[a], Jianjun Hu[a], Pengchong Liu[a], Dan Cui[a], Hongqin Di[a], Shucai Wu[a]*

[a] Hebei Chest Hospital, Shijiazhuang, 050041, China

*To whom correspondence should be addressed.

Tel: +86-311-86911298

Email: wushc2009@sina.com

SUPPLEMENTARY FIGURES

FIGURE LEGENDS

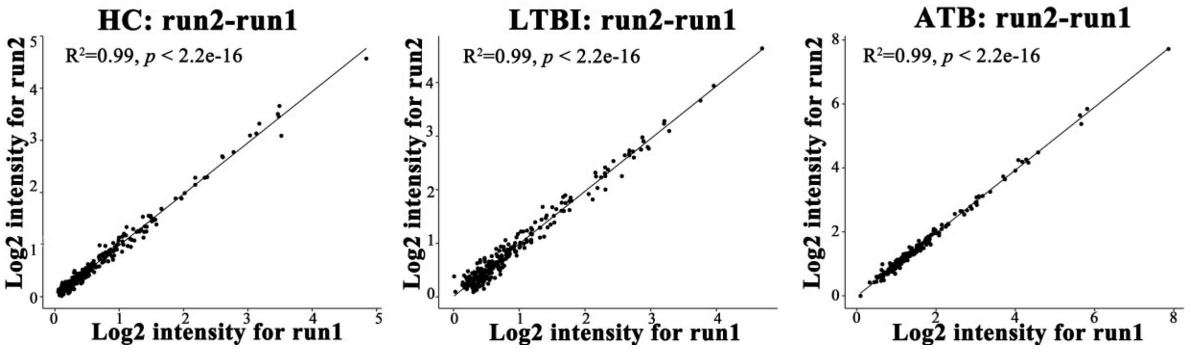

Supplementary Figure 1. Quality control testing of the secreted protein microarray indicates a high degree of reproducibility. Intensity scatter plots of two representative analyses of serum samples from HC groups, LTBI groups and ATB groups (selected randomly). To examine the reproducibility of experimental results, the correlation between the intensities of quantified proteins on any two runs were analyzed. The average Pearson correlation coefficient (R2) was 0.99, suggesting the reliability and reproducibility of the microarray experiments was high.

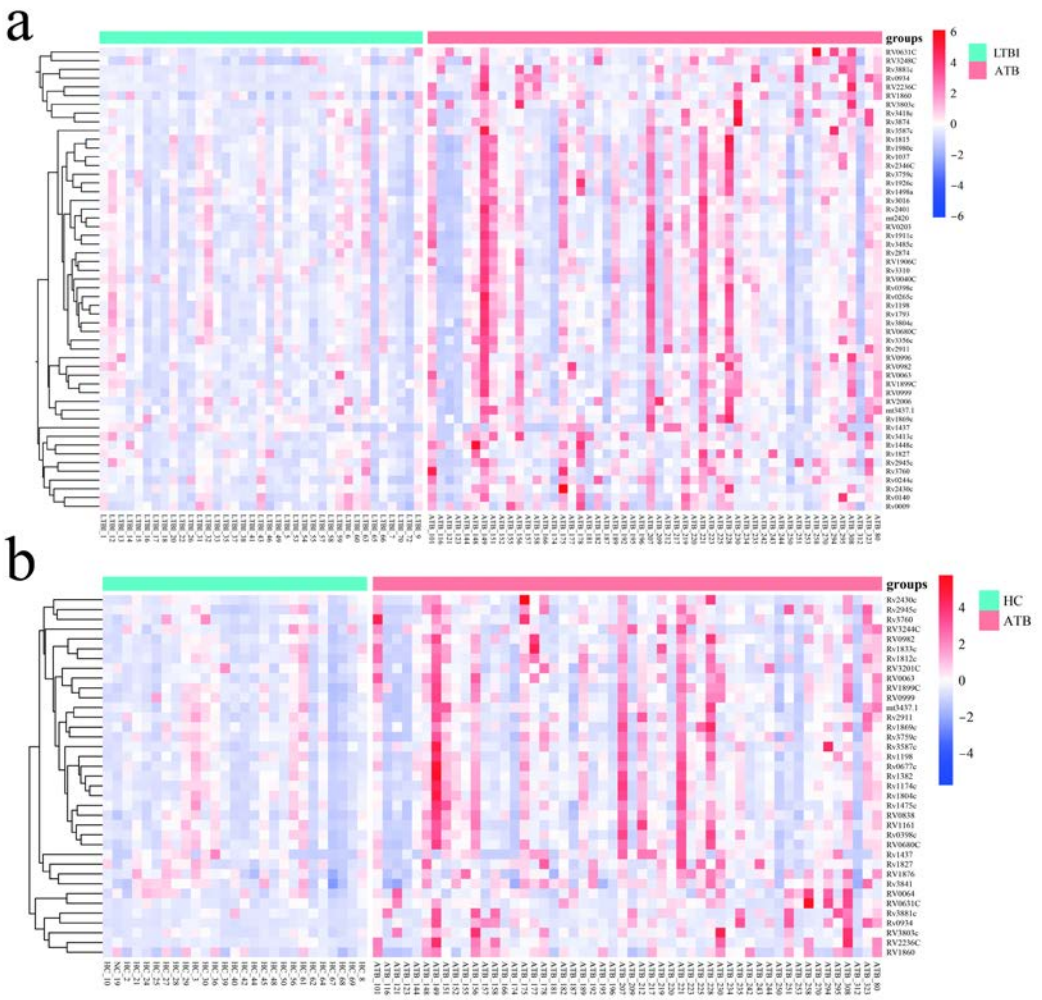

Supplementary Figure 2. Hierarchical clustering analysis of differential proteins. Hierarchical clustering analysis of differential proteins from the ATB and LTBI comparison **(a)**, ATB and HC comparison **(b)**. The color bar representing the signal intensity of the antigens is shown on the right. The heatmap was draw using the ‘pheatmap’ package (v1.0.12).
